# Unraveling Quantum Annealers using Classical Hardness

**DOI:** 10.1038/srep15324

**Published:** 2015-10-20

**Authors:** Victor Martin-Mayor, Itay Hen

**Affiliations:** 1 Departamento de Física Teórica I, Universidad Complutense, 28040 Madrid, Spain; 2 Instituto de Biocomputación y Física de Sistemas Complejos (BIFI), Zaragoza, Spain; 3 Information Sciences Institute, University of Southern California, Marina del Rey, California 90292, USA; 4 Center for Quantum Information Science & Technology, University of Southern California, Los Angeles, California 90089, USA

## Abstract

Recent advances in quantum technology have led to the development and manufacturing of experimental programmable quantum annealing optimizers that contain hundreds of quantum bits. These optimizers, commonly referred to as ‘D-Wave’ chips, promise to solve practical optimization problems potentially faster than conventional ‘classical’ computers. Attempts to quantify the quantum nature of these chips have been met with both excitement and skepticism but have also brought up numerous fundamental questions pertaining to the distinguishability of experimental quantum annealers from their classical thermal counterparts. Inspired by recent results in spin-glass theory that recognize ‘temperature chaos’ as the underlying mechanism responsible for the computational intractability of hard optimization problems, we devise a general method to quantify the performance of quantum annealers on optimization problems suffering from varying degrees of temperature chaos: A superior performance of quantum annealers over classical algorithms on these may allude to the role that quantum effects play in providing speedup. We utilize our method to experimentally study the D-Wave Two chip on different temperature-chaotic problems and find, surprisingly, that its performance scales unfavorably as compared to several analogous classical algorithms. We detect, quantify and discuss several purely classical effects that possibly mask the quantum behavior of the chip.

Interest in quantum computing originates in the potential of quantum computers to solve certain computational problems much faster than is possible classically, due to the unique properties of Quantum Mechanics^1^,^2^. The implications of having at our disposal reliable quantum computing devices are obviously tremendous. The actual implementation of quantum computing devices is however hindered by many challenging difficulties, the most prominent being the control or removal of quantum decoherence^3^. In the past few years, quantum technology has matured to the point where limited, task-specific, non-universal quantum devices such as quantum communication systems, quantum random number generators and quantum simulators, are being built, possessing capabilities that exceed those of corresponding classical computers.

Recently, a programmable quantum annealing machine, known as the D-Wave chip^4^, has been built whose goal is to minimize the cost functions of classically-hard optimization problems presumably by adiabatically quenching quantum fluctuations. If found useful, the chip could be regarded as a prototype for general-purpose quantum optimizers, due to the broad range of hard theoretical and practical problems that may be encoded on it.

The capabilities, performance and underlying physical mechanism driving the D-Wave chip have generated fair amounts of curiosity, interest, debate and controversy within the Quantum Computing community and beyond, as to the true nature of the device and its potential to exhibit clear “quantum signatures”. While some studies have concluded that the behavior of the chip is consistent with quantum open-system Lindbladian dynamics^5^, or indirectly observed entanglement^6^,^7^, other studies contesting these^8^,^9^, pointed to the existence of simple, purely classical, models capable of exhibiting the main characteristics of the chip.

Nonetheless, the debate around the quantum nature of the chip has raised several fundamental questions pertaining to the manner in which quantum devices should be characterized in the absence of clear practical “signatures” such as (quantum) speedups^10^,^11^,^12^. Since quantum annealers are meant to utilize an altogether different mechanism for solving optimization problems than traditional classical devices, methods for quantifying this difference are expected to serve as important theoretical tools while also having vast practical implications.

Here, we propose a method that partly solves the above question by providing a technique to characterize and quantitatively measure how detrimental classical effects are to the performance of quantum annealers. This is done by studying the algorithmic performance of quantum annealers on sets of optimization problems possessing quantifiable, varying degrees of “thermal” or “classical” hardness, which we also define for this purpose. To illustrate the potential of the proposed technique, we apply it to the experimental quantum annealing optimizer, the D-Wave Two (DW2) chip.

We observe several distinctive phenomena that reveal a strong correlation between the performance of the chip and classical hardness: i) The D-Wave chip’s typical time-to-solution (*t*_s_) as a function of classical hardness scales differently, in fact worse, than that of thermal classical algorithms. Specifically, we find that the chip does very poorly on problem instances exhibiting a phenomenon known as “temperature chaos”. ii) Fluctuations in success probability between programming cycles become larger with increasing hardness, pointing to the fact that encoding errors become more pronounced and destructive with increasing hardness. iii) The success probabilities obtained from harder instances are affected more than easy instances by changes in duration of the anneals.

Analyzing the above findings, we identify two major probable causes for the chip’s observed “sub-classical” performance, namely i) that its temperature may not be low enough, and ii) that encoding errors become more pronounced with increasing hardness. We further offer experiments and simulations designed to detect and subsequently rectify these so as to enhance the capabilities of the chip.

## Classical Hardness, temperature chaos and parallel tempering

In order to study the manner in which the performance of quantum annealers correlates with ‘classical hardness’, it is important to first accurately establish the meaning of *classical hardness*. For that purpose, we refer to spin-glass theory^13^, which deals with *spin glasses*—disordered, frustrated spin systems that may be viewed as prototypical classically-hard (also called NP-hard) optimization problems, that are so challenging that specialized hardware has been built to simulate them^14^,^15^,^16^.

Currently, the (classical) method of choice to study general spin-glass problems is Parallel Tempering (PT, also known as ‘exchange Monte Carlo’)^17^,^18^. PT is a refinement of the celebrated yet somewhat outdated Simulated Annealing (SA) algorithm^19^, that finds optimal assignments (i.e., the ground-state configurations) of given discrete-variable cost functions. It is therefore only natural to make use of the performance of PT to characterize and quantify classical hardness.

**Figure 1:**
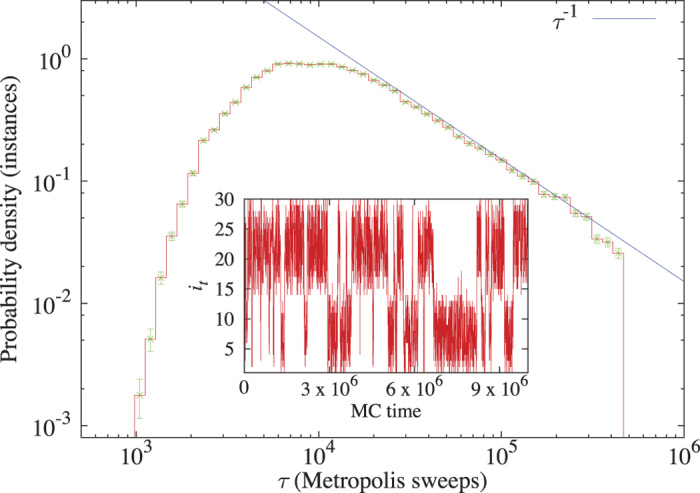
Probability distribution of mixing times *τ* over random *J* = ±1 ‘Chimera’ instances as extracted from the PT random-walk on the temperature grid. The solid line is a linear fit to the tail of the distribution, implying the existence of rare instances with very long mixing times (note that a *τ*^−1^ tail is not strictly integrable, hence this specific power law decay should only be regarded as a finite-size approximation). Inset: Example of a temperature random walk for an instance with 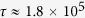. Considering one of 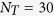 copies of the system, at any given Monte Carlo time *t*, the copy’s temperature is 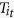. In this example, the replica has visited each temperature several times, pointing to the fact that the simulation time is longer than the mixing time *τ*.

In PT simulations, one considers *N*_*T*_ copies of an *N*-spin system at temperatures 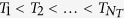, where each copy undergoes Metropolis spin-flip updates independently of other copies. In addition, copies with neighboring temperatures regularly attempt to swap their temperatures with probabilities that satisfy detailed balance^20^. In this way, each copy performs a temperature random-walk (see inset of Fig 1). At high temperatures, free-energy barriers are easily overcome, allowing for a global exploration of configuration space. At lower temperatures on the other hand, the local minima are explored in more detail. A ‘healthy’ PT simulation requires an unimpeded temperature flow: The total length of the simulation should be longer than the temperature ‘mixing time’ *τ*^21^,^22^ The time *τ* may be thought of as the average time it takes each copy to fully traverse the temperature mesh, indicating equilibration of the simulation. Therefore, instances with large *τ* are harder to equilibrate, which motivates our definition of the mixing time *τ* as the *classical hardness* of a given instance.

**Figure 2:**
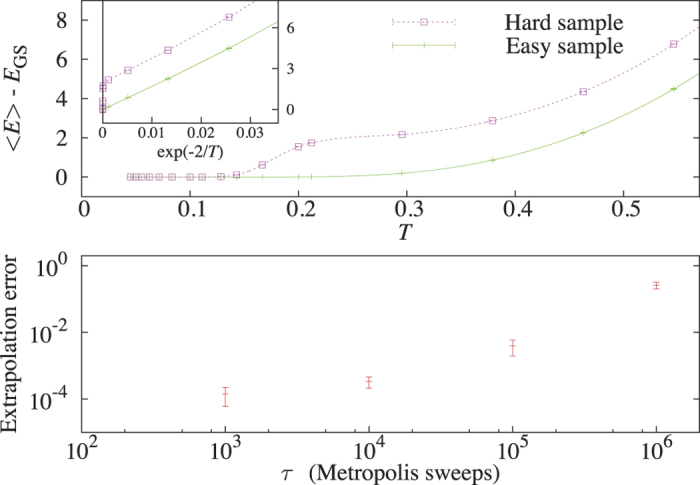
Top: Energy above the ground-state energy as a function of temperature for two randomly chosen instances. The mixing times of the two instances are 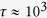 (“easy”) and 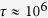 (“hard”). Unlike the easy sample, the hard sample exhibits “temperature chaos”: Upon lowering the temperature, the energy decreases at first in a gradual manner, however at 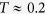 there is a sudden drop indicating that a different set of minimizing configurations has been visited. Inset: Main panel’s data vs. 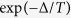 (where Δ = 2 is the excitation gap). A linear behavior is expected if the system can be described as a gas of non-interacting excitations over a local energy minimum. For the easy sample, this local minimum is a ground state. On the other hand, for instances displaying TC above their chaos temperature, the local-minimum energy is higher than the ground-state’s. Bottom: *τ*-dependence of the median systematic error (for each *τ*-generation) of a *T*→0 extrapolation of the total energy. For each instance, we extrapolated the *T* = 0.2, 0.3 data linearly in 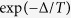 and compared this extrapolation with the actual ground-state energy.

Despite the popularity of PT, it has also become apparent that not all the spin-glass problems can be efficiently solved by the algorithm^22^,^23^. The reason is a phenomenon that has become known as *Temperature Chaos* (TC). TC^23^,^24^,^25^,^26^,^27^,^28^,^29^,^30^,^31^,^32^,^33^,^34^,^35^,^36^,^37^,^38^ consists of a sequence of first-order phase transitions that a given spin-glass instance experiences upon lowering its temperature, whereby the dominant configurations minimizing the free energy above the critical temperatures are vastly different than those below them (Fig. 2 depicts such a phase transition that is ‘rounded’ due to the finite size of the system). A given instance may experience zero, one or more transitions at random temperatures, making the study of TC excruciatingly difficult^23^,^36^,^37^,^38^. Such TC transitions hinder the PT temperature flow, significantly prolonging the mixing time *τ*. In practice^23^, it is found that for small systems the large majority of the instances do not suffer any TC transitions and are ‘easy’ (i.e., they are characterized by short mixing times). However, for a minor fraction of them, *τ* turns out to be inordinately large. Moreover, the larger the system is, the larger the fraction of long-*τ* samples becomes. In the large *N* limit, these are the short-*τ* samples that become exponentially rare in *N*^23^,^34^. This provides further motivation for studying TC instances of optimization problems on moderately small experimental devices (even if these problems are rare).

## Temperature chaos and quantum annealers

With the advent of quantum annealers, which presumably offer non-thermal mechanisms for finding ground states, it has become only natural to ask whether quantum annealers can be used to solve ‘TC-ridden’ optimization problems faster than classical techniques such as PT. In this context, the question of how the performance of quantum annealers depends on the ‘classical hardness’ becomes of fundamental interest: If indeed quantum annealers exploit quantum phenomena such as tunneling to traverse energy barriers, one may hope that they will not be as sensitive to the thermal hardness (as defined above) of the optimization problems they solve. As we shall see next, having a practical definition for classical hardness allows us to address the above questions directly.

To illustrate this, in what follows we apply the ideas introduced above to the DW2 quantum annealing optimizer. We accomplish this by first generating an ensemble of instances that are directly embeddable on the DW2 ‘Chimera’ architecture [the reader is referred to the Supplementary Information (SI) for a detailed description of the Chimera lattice and the D-wave chip and its properties]. The chip on which we perform our study is an array of 512 superconducting flux qubits of which only 476 are functional, operating at a temperature of ~15 mK. The DW2 chip is designed to solve a very specific type of problems, namely, Ising-type optimization problems, by adiabatically transitioning the system Hamiltonian from an initial transverse-field Hamiltonian to a final classical programmable cost function of a typical spin glass. The latter is given by the Ising Hamiltonian:


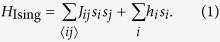


The Ising spins, 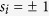 are the variables to be optimized over, and the sets 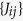 and 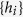 are programmable parameters of the cost function. Here, 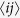 denotes a sum over all the active edges of the Chimera graph. For simplicity, we conduct our study on randomly-generated problem instances with *h*_*i*_ = 0 and random, equiprobable *J*_*ij*_ = ±*J* couplings (in our energy units *J* = 1).

Initially, it is not clear whether the task of finding thermally-hard instances on the Chimera is feasible. While on the one hand TC has been observed in spin-glasses on the square lattice^39^, which has the same spatial dimension, *D* = 2, as the Chimera^40^, it has also been found that typical Chimera-embeddable instances are easy to solve 11,12,40. As discussed above, system size plays a significant role in this context, as an *N*~512-spin Chimera may simply be too small to have instances exhibiting TC (for instance, on the square lattice one needs to reach 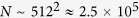 spins for TC to be the rule rather than the exception^39^). Taking a brute-force approach to resolve this issue, we generated ~80,000 random problem instances (each characterized by a different set of 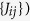, analyzing each one by running them on a state-of-the-art PT algorithm until equilibration is reached (see the SI). This allowed for the calculation of the instances’ classical hardness, namely their temperature mixing times *τ* (for more details, see **Methods**, below). The resulting distribution of *τ* over the instances is shown in Fig. 1. While most instances equilibrate rather quickly (after some 10^4^ Monte Carlo steps), we find that the distribution has a ‘tail’ of hard samples with *τ* > 10^6^ revealing that hard instances, although rare, do exist (we estimate that 2 samples in 10^4^ have *τ* > 10^7^).

To study the DW2 chip, we grouped together instances with similar classical hardness, i.e., similar mixing times, 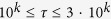 for *k* = 3, 4, 5, 6 and 7. For each such ‘generation’ of *τ*, we randomly picked 100 representative instances for running on the chip (only 14 instances with *k* = 7 were found). As a convergence test of PT on the selected instances, we verified that the ground-state energies reached by PT are the true ones by means of an exact solver.

At the purely classical level, we found, as anticipated^23^, that classically hard instances differ from easy instances from a thermodynamic point of view as well. Specifically, large *τ* instances were found to exhibit sharp changes in the average energy at random critical temperatures, consistently with the occurrence of TC (see Fig. 2). For such instances, the true ground states are present during the simulations only below the TC critical temperatures. As the inset shows, the larger *τ* is, the lower these critical temperatures typically are. Furthermore, classically-hard instances were found to differ from easier ones in terms of their energy landscape: While for easy instances minimally-excited states typically reside only a few spin flips away from ground state configurations, for classically hard instances, this is not the case (see the SI).

## Effects of temperature chaos on the “D-Wave Two” chip

Having sorted and analyzed the randomly-generated instances, we turned to experimentally test the performance of the D-Wave chip on these (for details see **Methods** and SI). Our experiments consisted of programming the chip to solve each of the 414 instances over a dense mesh of annealing times in the available range of 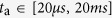. The number of attempts, or anneals, that each instance was run for each choice of *t*_a_ ranged between 10^5^ and 10^8^. By calculating the success probability of the annealer on each instance and annealing time, a typical time-to-solution *t*_s_ was obtained for each hardness-group, or ‘*τ*-generation’ (see **Methods**). Interestingly, we found that for easy samples (*τ* = 10^3^) the success probability depends only marginally on *t*_a_, pointing to the annealer reaching its asymptotic performance on these. As instances become harder, the sensitivity of success probability to *t*_a_ increases significantly. Nonetheless, for all hardness groups, the typical *t*_s_ is found to be shortest at the minimally-allowed annealing time of *t*_a_ = 20 *μ*s (see the SI for a more detailed discussion).

**Figure 3:**
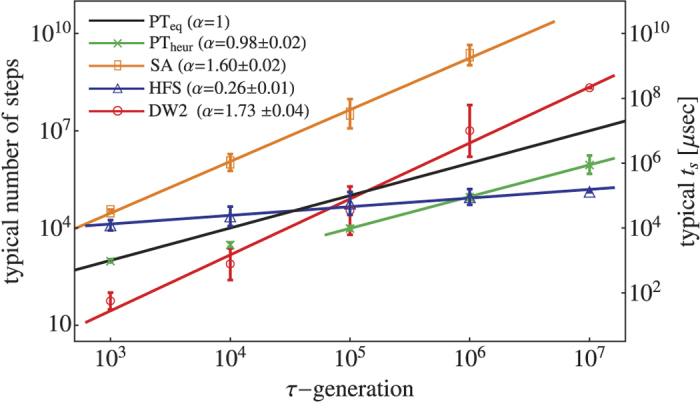
Dependence of typical time to solution *t*_s_ of the examined optimization algorithms on mixing time *τ*, the classical hardness parameter. Here, PT_eq_ denotes time to equilibration which by definition scales linearly with *τ*, and PT_heur_ denotes PT functioning as a heuristic solver in which case the time to solution is the number of Monte-Carlo steps to first encounter of a minimizing configuration. The *t*_s_ of the SA algorithm scales as 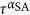, with 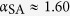 whereas that of the classical non-thermal HFS algorithm (measured in *μ*s) scales as 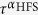, with 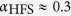. The *t*_s_ for the DW2 chip (measured in *μ*s) scales as 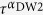 with 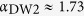 (we note the missing error bars on the 10^7^ DW2 data point, which is due to insufficient statistics).

The main results of our investigation are summarized in Fig. 3 depicting the typical time to solution *t*_s_ of the DW2 chip (averaged over instances of same hardness groups, see **Methods**) as a function of classical hardness, or ‘*τ*-generation’. As is clear from the figure, the performance of the chip was found to correlate strongly with the ‘thermal hardness’ parameter, indicating the significant role thermal hardness plays in the annealing process. Interestingly, the response of the chip was found to be affected by thermal hardness even more than PT, i.e., more strongly than the classical thermal response: While for PT the time-to-solution scales as *t*_s_ ~ *τ*, the scaling of the D-Wave chip was found to scale as 
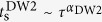
, with 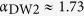. This scaling is rather surprising given that for quantum annealers to perform better than classical ones, one would expect these to be less susceptible to thermal hardness, not more. Nonetheless, it is clear that the notion of classical hardness is very relevant to the D-wave chip.

To complete the picture, we have also tested our instances with two other classical algorithms. The first is Simulated Annealing (SA) which has recently become a popular benchmarking algorithm against which D-Wave chips are tested^10^,^11^,^12^ (see **Methods**), and the second is the Hamze-de Freitas-Selby (HFS) algorithm^41^,^42^ which is the fastest classical algorithm to date for Chimera-type instances. As expected, SA, which is a thermal method, is found to be sensitive to TC, even more than PT, yielding 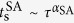 with 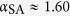. Even though the estimate of 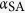 presumably slightly depends on our choice of success metric and the details of the algorithm, its susceptibility to classical hardness is nonetheless very evident.

The HFS algorithm is a ‘non-thermal’ algorithm (i.e., it does not make use of a temperature parameter). Nonetheless, here too we have found the concept of classical hardness to be very relevant. For the HFS algorithm, we find a scaling of 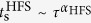 with 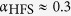, implying that the algorithm is significantly less susceptible to thermal hardness than PT (it is worth pointing out that the typical runtime for the HFS algorithm on the hardest, *τ* = 10^7^, group problems was found to be ~0.5 s on an Intel Xeon CPU E5462 @ 2.80 GHz, which to our knowledge makes these the hardest known Chimera-type instances to date).

## Analysis of findings

The above somewhat less-than-favorable performance of the experimental D-wave chip on thermally-hard problems is not necessarily a manifestation of the intrinsic limitations of quantum annealing, i.e., it does not necessarily imply that the ‘quantum landscape’ of the tested problems is harder to traverse than the classical one (although this may sometimes be the case^43^,^44^). A careful analysis of the results suggests in fact at least two different more probable ‘classical’ causes for the performance of the chip.

First, as already discussed above and is succinctly captured in Fig. 2, temperature is expected to play a key role in DW2 success on instances exhibiting TC. This is because for these, the ground state configurations minimize the free energy only below the lowest critical TC temperature. Even though the working temperature of the DW2 chip is rather low, namely ~15 mK, the crucial figure of merit is the ratio of coupling to temperature *T*/*J* [recall Eq. (1)]. Although the nominal value for the chip is 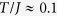 (calculated with reference to the classical Hamiltonian at the end of the anneal), any inhomogeneity of the temperature across the chip may render the ratio higher, possibly driving it above typical TC critical temperatures. (We refer to the *physical* temperature of the chip. However, non-equilibrium systems, e.g., supercooled liquids or glasses, can be characterized by *two* temperatures^45^: On the one hand, the physical temperature *T* which rules fast degrees of freedom that equilibrate. On the other hand, the ‘effective’ temperature refers to slow degrees of freedom that remain out of equilibrium. We are currently investigating whether or not the DW2 chip can analogously be characterized by two such temperatures.)

Another possible cause for the above scaling may be due to the analog nature of the chip. The programming of the coupling parameters 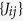 and magnetic fields 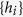 is prone to statistical and systematic errors (also referred to as intrinsic control errors, or ICE). The couplings actually encoded in DW2 are 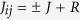, where 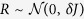 is a random error (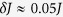, according to the manufacturer of the chip). Unfortunately, even tiny changes in coupling values are known to potentially change the ground-state configurations of spin glasses in a dramatic manner^37^,^46^,^47^,^48^. We refer to this effect as ‘coupling chaos’ (or *J*-chaos, for short). For an *N*-bit system, *J*-chaos seems to become significant for 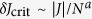. Empirically^37^,^48^ 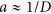, *D* being the spatial dimension of the system. Note, however, that these estimates refer only to typical instances and small *N* whereas the assessment of the effects of *J*-chaos on thermally-hard instances remains an important open problem for classical spin glasses.

**Figure 4:**
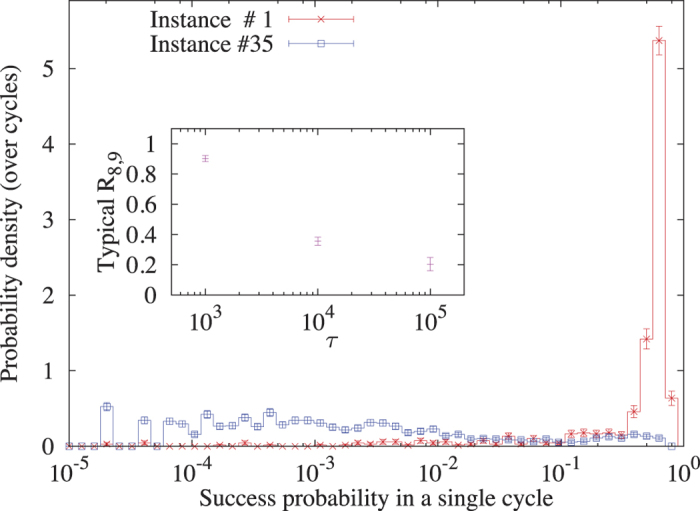
Empirical evidence for the absence/presence of ‘*J*-chaos’. Probability density of success probability of a single cycle, *p*, over different programming cycles. The probability densities are plotted here for two easy (*τ* = 10^3^) instances (here, *t*_a_ = 20 *μ*s and the number of anneals per programming cycle is *X* = 49500, see **Methods**). Instance #1 (662 cycles) is typical in this *τ*-generation with success probability *p* ~ 1 in the majority of the programming cycles. On the other hand, instance #35 (1624 cycles) suffers from strong *J*-chaos: Even though the probability of finding *p* ~ 0.1 in some of the programming cycles is not negligible, most cycles are significantly less successful, e.g., the median *p* is 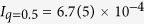. **Inset:** Typical ratio of the 80th percentile probability to the 90th percentile probability, namely 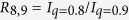 (see text) as a function of *τ* (for *τ* ≥ 10^6^ and 10^7^, *R*_8,9_ was not computed due to extremely low success probabilities). Smaller ratios indicate larger fluctuations in success probabilities.

Here, we empirically quantify the effects of *J*-chaos by taking advantage of the many programming cycles and gauge choices each instance has been annealed with (typically between 200 and 2000). Calculating a success probability *p* for each cycle, we compute the probability distribution of *p* over different cycles for each instance^23^,^38^. We find that while for some instances *p* is essentially insensitive to programming errors, for other instances (even within the same thermal hardness group), *p* varies significantly, spanning several orders of magnitude. This is illustrated in Fig. 4 which presents some results based on a straightforward percentile analysis of these distributions. For instance #1 in the figure, the 80th percentile probability is 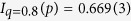, whereas the probability at the 90th percentile is 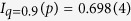. Hence the ratio 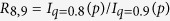 is close to one. Conversely, for instance #35, the values are 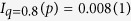, 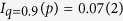 and the ratio is 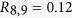, i.e., the success probability drops by an order of magnitude. The inset of Fig. 4 shows the typical ratio 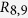 as a function of classical hardness, demonstrating the strong correlation between thermal hardness and the devastating effects of *J*-chaos caused by ICE, namely that the larger *τ* is, the more probable it is to find instances for which *p* varies wildly between programming cycles.

## Discussion

We have devised a method for quantifying the susceptibility of quantum annealers to classical effects by studying their performance on sets of instances characterized by different degrees of thermal hardness, which we have defined for that purpose as the mixing (or equilibration) time *τ* of classical thermal algorithms (namely, PT) on these. We find that the 2D-like Chimera architecture used in the D-Wave chips does give rise to thermally very-hard, albeit rare, instances. Specifically, we have found samples that exhibit temperature chaos, and as a such have very long mixing times, i.e., they are classically exceptionally hard to solve.

We demonstrated that ‘temperature chaos’, or TC, is an inherent property of the (classical) free-energy landscape of certain (hard) optimization problem instances, and is expected to universally hinder the performance of *any* heuristic optimization algorithm.

Applying our method to an experimental quantum annealing optimizer, the DW2 chip, we have found that its performance is more susceptible to changes in thermal hardness than classical algorithms. This is in contrast with the performance of the best-known state-of-the-art classical solver on Chimera graphs, the ‘non-thermal’ HFS algorithm, which scales (unsurprisingly) better. Our results are not meant to suggest that the DW2 chip is not a quantum annealer, but rather that its performance is greatly impeded by much-undesired classical effects.

We have identified and quantified two probable causes for the observed behavior: A possibly too high temperature, or more probably, *J*-chaos, the random errors stemming from the digital-to-analog conversion in the programming of the coupling parameters. One may hope that the scaling of current DW2 chips would significantly improve if one or both of the above issues are resolved. Clearly, lowering the temperature of the chip and/or reducing the error involved in the programming of its parameters are both technologically very ambitious goals, in which case error correcting techniques may prove very useful^49^. We believe that quantum Monte Carlo simulations of the device will be instrumental to the understanding of the roles that temperature and magnitude of programming errors play in the performance of the chip (and of its classical counterparts). In turn, this will help sharpening the most pressing technological challenges facing the fabrication of these and other future quantum optimizing devices, paving the way to obtaining long-awaited insights as to the difference between quantum and classical hardness in the context of optimization. We are currently pursuing these approaches.

## Methods

### Computation of the mixing time *τ*

Because we follow ref. 22 we just briefly summarize here the main steps of the procedure. Considering one of the *N*_*T*_ system copies in the PT simulation, let us denote the temperature of copy *i* at Monte Carlo time *t* by 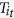, where 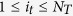 (see inset of Fig. 1). At equilibrium, the probability distribution for *i*_*t*_ is uniform (namely, 1/*N*_*T*_) hence the exact expectation value of *i*_*t*_ is 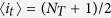. From the general theory of Markov Chain Monte Carlo^20^, it follows that the equilibrium time-correlation function may be written as a sum of exponentially decaying terms:


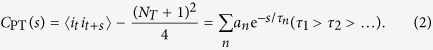


The mixing time *τ* is the largest ‘eigen-time’ *τ*_1_. We compute numerically the correlation function 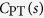 and fit it to the decay of *two* exponential functions (so we extract the dominant time scale *τ* and a sub-leading time scale). The procedure is described in full in ref. 22.

### D-wave data acquisition and analysis

#### Data acquisition

In what follows we briefly summarize the steps of the experimental setup and data acquisition for the anneals performed on the 414 randomly-generated instances in the various thermal-hardness groups.

1. The *J*_*ij*_ couplings of each of the 414 instances have been encoded onto the D-Wave chip using many different choices of annealing times in the allowed range of 20 *μ*s ≤ *t*_a_ ≤ 20 *m*s.
2. For each instance and each choice of *t*_a_ the following process has been repeated hundreds to thousands of times:
  - First, a random ‘gauge’ has been chosen for the instance. A gauge transformation does not change the properties of the optimization problem but has some effect on the performance of the chip which follows from the imperfections of the device that break the gauge symmetry. The different gauges are applied by transforming the original instance 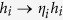, 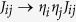, to the original cost function Eq. (1). The above gauge transformations correspond to the change 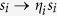 in configuration spin values. Here, the *N* gauge parameters 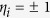 were chosen randomly.
  - The chip was then programmed with the gauge-transformed instance (inevitably adding programming bias errors, as mentioned in the main text).
  - The instance was then solved, or annealed, *X* times within the programming cycle/with the chosen gauge. We chose 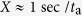, the maximally allowed amount.
  - After the *X* anneals were performed, the number of successes *Y*, i.e., the number of times a minimizing configuration had been found, was recorded. The probability of success for the instance, for that particular gauge/programming cycle and annealing time *t*_a_ was then estimated as 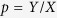. Note, that in cases where the probability of success is of the order of 1/*X*, the probability *p* will be a rather noisy estimate (see data analysis, below, for a procedure to mitigate this problem).
  - If the prefixed number of cycles for the current instance and *t*_a_ has not been reached, return to (2a) and choose a new gauge.

#### Data analysis and time-to-solution estimates

The analysis of the data acquisition process described above proceeded as follows.

1. For each instance and each annealing time, the total number of hits 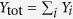 was calculated, where *i* sums over all the gauge/programming cycles. Denoting 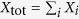 as the total number of annealing attempts, the probability of success for any particular instance and anneal time was then calculated as 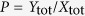.
2. The above probability was then converted into an average time-to-solution *t*_s_ for that instance and *t*_a_ according to *t*_s_ = *t*_a_/*P*, where the special case of *P* = 0 designates an estimate of an infinite *t*_s_, where in practice the true probability lies below the resolution threshold of 1/*X*_tot_.
3. A typical runtime for a hardness group was then obtained by taking the median over all minimal *t*_s_ values of all the instances in the group.

### Simulated Annealing (SA) runs

We employed the following protocol to find ground states for all 414 tested instances. Each annealing schedule was run 1024 times. The temperature range was chosen to be identical to that used in our Parallel Tempering simulations with a starting temperature of 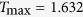, where the system was left to equilibrate for 1000 full-lattice Metropolis sweeps, and a final temperature of 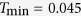 which is below typical TC critical points (see Fig. 2). The cooling schedule that was chosen is linear in 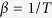 with the temperature decreasing after each full-lattice sweep. In the initial cooling rate, we sent the temperature from *T*_max_ down to *T*_min_ in 256 cooling steps. If the number of independent runs reaching a ground state at the end of each anneal was found to be less than one eighth of the total anneals (i.e., if less than 128 runs ended in a ground state), the number of cooling steps was doubled and the runs were restarted. The procedure was iterated until the “one-eighth success rate” criterion has been met. We then took the final number of cooling steps as a figure of merit for the success of the SA algorithm on that particular instance. Finally, we computed the median over the instances of each hardness group in order to obtain a characteristic time-to-solution *t*_s_. Interestingly, for several of the hardest problems, the “one-eighth success rate” criterion was not met even with 2 × 10^9^ cooling sweeps (40 instances in the *τ*-generation group 10^6^, as well as thirteen out of the fourteen instances in the *τ* ~ 10^7^ group).

## Additional Information

**How to cite this article**: Martin-Mayor, V. and Hen, I. Unraveling Quantum Annealers using Classical Hardness. *Sci. Rep.* **5**, 15324; doi: 10.1038/srep15324 (2015).

## Supplementary Material

Supplementary Information
