# Supplementary material for: Unraveling Quantum Annealers using Classical Hardness
Source: Sci Rep. 2015 Oct 20;5:15324. doi: 10.1038/srep15324 (PMC4611884; doi:10.1038/srep15324)
Supplement: Supplementary Information [file srep15324-s1.pdf]

Supplementary Information for
Unraveling Quantum Annealers using Classical Hardness

Victor Martin-Mayor
Departamento de Física Teórica I, Universidad Complutense, 28040 Madrid, Spain and
Instituto de Biocomputación y Física de Sistemas Complejos (BIFI), Zaragoza, Spain

Itay Hen
Information Sciences Institute, University of Southern California, Marina del Rey, California 90292, USA
(Dated: January 26, 2015)

INSTANCE GENERATION AND ANALYSIS

The Parallel Tempering simulations

We briefly outline the technical details of the Parallel Tempering (PT) simulations and subsequent analysis performed on the randomly generated instances. The reader is referred to Ref. [1] for further details.

To run the simulations, we employed multi-spin coding [2], a simple yet efficient form of parallel computation, that allows the simultaneous simulations of a large number of problem instances. The name *multi-spin* follows from the fact that our dynamic variables are binary, $s_i = \pm 1$, and thus can be coded in a single bit. Hence, one may code the i-th spin corresponding to M independent instances in a single M-bit computer word. Using streaming extensions, M can be as large as $M = 256$, nowadays.[1] The advantages of simulating in parallel 256 instances are obvious, if we want to study a *huge* number of problems in our quest for these rare instances displaying Temperature Chaos. In fact, we have simulated a total of $N_S = 303 \times 256 = 77568$ problem instances.

The temperature grid of the PT simulations consisted of $N_T = 30$ temperatures. Temperatures with indices $i = 13, 14, \ldots, N_T$ were evenly distributed in the range $0.21 \le T_i \le T_{\max} = 1.632$, while lower temperatures in the range $T_{\min} = 0.045 \le T_i \le 0.2$ (indices $i = 1, 2, \ldots, 12$) have been added in order to detect temperature chaos effects.[2] Ergodicity was maintained by the temperature-swap part of the PT.

For each instance, we ran four independent simulations (i.e., four replicas) per temperature, where an elementary Monte Carlo step consisted of 10 full lattice Metropolis sweeps, followed by a PT temperature swap attempt. Since the Chimera lattice is bipartite, Metropolis sweeps have been carried out on alternating partitions at each step.

The calculation of the mixing time τ was conducted in three stages, or rounds (see also Ref. [1]). In the first stage, all 303×256 instances were simulated for a total of 10^6 elementary Monte Carlo steps (i.e., each system was simulated for 10^7 full-lattice Metropolis sweep). At the end of each round the mixing time τ was computed (measured in units of full-lattice Metropolis sweeps). As can also be read off Fig. 1 of the main text, the first-round of simulations was adequate for the equilibration of most instances, namely, for problems of τ-generations $10^3, 10^4$ and 10^5. The second round of simulations was set to last 10 times longer, consisting of 10^7 elementary Monte Carlo steps, or 10^8 full-lattice Metropolis sweeps per system. These longer simulations were reserved only to the 1024 hardest instances corresponding to τ-generations of 10^6 or larger.[3] The 256 worse-scoring instances of these were further simulated for 10^8 elementary Monte Carlo steps (or 10^9 full-lattice Metropolis sweeps per system). This simulation lasted 10 days on our fastest CPU [Intel(R) Core(TM) i7-4770K CPU @ 3.50GHz]. From it, we obtained 14 extremely hard problem instances, belonging to τ-generation 10^7.

---

[1] Simulations were run on a standard Intel(R) Xeon(R) processors (E5-2690 0 @ 2.90GHz).

[2] In our simulations, the minimal temperature value $T_{\min} = 0.045$ corresponds effectively to zero temperature. This follows from the minimal energy gap of our instances being $\Delta = 2$ together with our use of a 64-bit (pseudo) random-number generator [3]. Setting $T_{\min}$ such that it obeys $2^{64} \times \mathrm{e}^{-2/T_{\min}} < 1$, our Metropolis simulations at $T_{\min}$ effectively become equivalent to $T = 0$ simulations.

[3] The criterion for selecting the hardest instances to qualify for subsequent rounds of simulations was done by assigning a figure of merit for each instance based on the performance of its $4 \times 30 = 120$ system copies. For each of the copies, the fraction of Monte Carlo time spent at the higher-temperatures regions, namely T_i with $i = 16, 17, \ldots, N_T$, was calculated where the figure of merit was chosen to be the smallest of these fractions, which was used as an indication for how trapped the instance is in the low-temperatures region.

Spin-overlap analysis

Here, we briefly extend the analysis of the energy landscapes of thermally-hard problems, initiated in Fig. 2 of the main text. To do so, we make use of the notion of 'spin-overlap', which plays a major role in any spin glass investigation (see, e.g., Ref. [4]). The spin overlap q between two N-spin configurations $\{s_i^a\}$ and $\{s_i^b\}$ is defined as

$$q = 1 - 2\frac{N_{a,b}}{N}\,, \tag{1}$$

where $N_{a,b}$ is the Hamming distance between the two configurations, i.e., the number of spins by which the two configurations *differ*. For two identical configurations we get $N_{a,b} = 0$ and $q = 1$ while for two configurations differing by a global spin flip (i.e., $s_i^a = -s_i^b$ for all i) we have $N_{a,b} = N$ and $q = -1$. Since the instances we study all have $h_i = 0$, their Hamiltonians possess a global bit-flip symmetry. For any configuration $\{s_i^a\}$, its spin-reversed configuration $\{s_i^b\} = -\{s_i^a\}$ has the same energy. We have therefore chosen to consider the more informative measure of the absolute value of the overlap, $|q|$. In this case, the maximum meaningful Hamming distance between any two configurations is $N/2$.

We utilized spin overlaps to investigate the energy landscapes of the various instances in the following way. During the PT simulations, 12000 spin configurations of each instance were stored on disk, corresponding to 100 evenly spaced check-points in Monte Carlo time where every check-point contains $4 \times 30 = 120$ configurations. Of those, ground-state (GS) configurations[4] and minimally-excited (ES) states were picked out for further analysis. Our analysis consisted of examining the probability distributions of overlaps between (i) randomly chosen GS configurations and randomly chosen ES configurations [GS-ES, solid blue curves in Fig. 1 of Extended Data (ED)] and (ii) pairs of randomly chosen GS configurations (GS-GS, dashed red curves in ED-Fig. 1). Two extremal cases were encountered. For easy instances (ED-Fig. 1–top), the probability density for GS-GS overlaps is very similar to the probability density for GS-ES overlaps. This is expected in cases where ES states are trivially connected to GS states via one spin-flip. Conversely, for hard instances (ED-Fig. 1–bottom), the probability densities for GS-GS overlaps and for GS-ES overlaps differ significantly, indicating that the vast majority of the ES states encountered during the simulations are not trivially connected to typical GS configurations but are rather very distant from them.

The top and bottom panels of ED-Fig. 1 describe only two representative instances, however the above depiction was also found to be valid in the general case, as is confirmed by calculation of the typical $|q|$ for all the instances in each of the hardness groups.[5] As the inset of ED-Fig. 1 shows, the harder instances are, the further away minimally excited states are from ground states.

THE D-WAVE TWO CHIP

The Chimera

The Chimera graph of the D-Wave Two (DW2) chip used in this study is shown in ED-Fig. 2. The chip is an 8×8 array of unit cells where each unit cell is a balanced $K_{4,4}$ bipartite graph of superconducting flux qubits. In the ideal Chimera graph the degree of each (internal) vertex is 6. On our chip, only a subset of 476 qubits, is functional. The temperature of the device $\sim$ 15mK.

The chip is designed to solve a very specific type of problems, namely, Ising-type optimization problems where the cost function is that of the Ising Hamiltonian [see Eq. (1) of the main text]. The Ising spins, $s_i = \pm 1$ are the variables to be optimized over and the sets $\{J_{ij}\}$ and $\{h_i\}$ are the programmable parameters of the cost function. In addition, $\langle ij \rangle$ denotes a sum over all active edges of the Chimera graph.

[4] For a $J = \pm 1$ spin-glass, the ground state is typically highly degenerate even in two dimensions [5]. Empirically, we find that this high-degeneracy is also present on the 2D-like Chimera [6].

[5] In order to define a typical $|q|$, we follow a two-steps procedure. First, we obtain a typical $|q|$ for each instance by computing the median overlap [e.g. for the easy instance in the top panel of ED-Fig. 1 med($|q|$) = 0.918, while for the hard instance in the bottom panel med($|q|$) = 0.25]. Second, we average over all the instances in a τ-generation, by computing the median over the instance medians.

The D-Wave Two annealing schedule

The DW2 performs the annealing by implementing the time-dependent Hamiltonian

$$H(t) = -A(t)\sum_i \sigma_i^x + B(t)H_{\text{Ising}}\,, \tag{2}$$

with $t \in [0, t_{\text{a}}]$ where the allowed range of annealing times t_{a}, due to engineering restrictions, is between 20μs and $20ms$. The annealing schedules $A(t)$ and $B(t)$ used in the device are shown in ED-Fig. 3. There are four annealing lines, and their synchronization becomes harder for faster annealers. The filtering of the input control lines introduces some additional distortion in the annealing control.

Gauge averaging on the D-Wave device

Calibration inaccuracies stemming mainly from the digital to analog conversions of the problem parameters, cause the couplings J_{ij} and h_i realized on the DW2 chip to be slightly different from the intended programmed values (with a typical $\sim 5\%$ variation). Therefore, instances encoded on the device will be generally different from the intended instances. Additionally, other, more systematic biases exist which cause spins to prefer one orientation over another regardless of the encoded parameters. To neutralize these effects, it is advantageous to perform multiple annealing rounds (or 'programming cycles') on the device, where each such cycle corresponds to a different encoding or 'gauge' of the same problem instance onto the couplers of the device [7]. To realize these different encodings, we use a gauge freedom in realizing the Ising spin glass: for each qubit we can freely define which of the two qubits states corresponds to $s_i = +1$ and $s_i = -1$. More formally this corresponds to a gauge transformation that changes spins $s_i \to \eta_i s_i$, with $\eta_i = \pm 1$ and the couplings as $J_{ij} \to \eta_i \eta_j J_{ij}$ and $h_i \to \eta_i h_i$. The simulations are invariant under such a gauge transformation, but due to calibration errors which break the gauge symmetry, the results returned by the DW2 are not.

Performance of the DW2 chip as a function of annealing time

Since the DW2 chip is a putative quantum annealer, it is only natural to ask how its performance, namely, the typical time-to-solution t_{s} it yields, depends on annealing time t_{a}. Ideally, the longer t_{a} is, the better the performance we expect [8]. However, in practice, decohering interactions with the environment are present which become more pronounced with longer running times of the annealing process. It is therefore plausible to assume that there is an optimal t_{a} for which t_{s} is shortest [7].

Analysis of the dependence of success probabilities, or equivalently times to solution, on annealing time is found to be heavily blurred, or masked, by fluctuations in the success probability between different programming cycles. These fluctuations, which become more pronounced for harder instances, stem from the noisy encoding of the instance parameters already discussed above. Any meaningful analysis of the dependence of success probability on annealing time must therefore successfully average out these effects therefore requires many rounds of anneals. Unfortunately (see data acquisition in **Methods**) the number of annealing attempts X for a given DW2 programming cycle is proportional to $1/t_{\text{a}}$, ranging from $X^{t_{\text{a}}=20\mu s} = 49500$, to $X^{t_{\text{a}}=20ms} = 49$. As a consequence, the minimal success probability that can be measured from a $t_{\text{a}} = 20ms$ programming cycle is just $p_{\text{min}}^{20ms} = 1/49 \approx 0.02$. While this limitation is not serious for easy instances (i.e., those of the $\tau = 10^3$ group), for hard instances a typical success probability p is much smaller than 0.02. This problem can be alleviated by running the DW2 chip on the same instance (and t_{a}) multiple number of times (with a different gauge for each cycle, but with t_{a} held fixed), and then averaging the resulting p over programming cycles. In this way, the minimal success probability that can be measured is $1/(XN_{\text{cycles}})$. For N_{cycles} in the hundreds typically, typical numbers for the minimal measurable success probability were

$$p_{\text{min}}^{20\mu\text{s}} \approx 6.5 \times 10^{-8}\,, \quad p_{\text{min}}^{200\mu\text{s}} \approx 3.3 \times 10^{-6}\,, \quad p_{\text{min}}^{2\text{ms}} \approx 4 \times 10^{-5} \text{ and } p_{\text{min}}^{20\text{ms}} \approx 5.1 \times 10^{-4}\,.$$

However, we have found that the above minimal-probability thresholds are still too high for our hardest problems, $\tau \geq 10^6$. To overcome this problem, we groups the success probabilities into annealing-time windows:

$$20\mu\text{s} \leq \frac{t_{\text{a}}}{10^k} < 60\mu\text{s}\,, \quad \text{and} \quad 60\mu\text{s} \leq \frac{t_{\text{a}}}{10^k} < 200\mu\text{s}\,, \quad \text{for } k = 0, 1, 2. \tag{3}$$

(In the largest-time interval, $k = 2$ above, we also included the $t_{\rm a} = 20$ms data).

At this point, the success probability needs to be averaged over the problem instances of a given τ-generation. Given the extreme problem-to-problem fluctuations, percentiles have been calculated. In ED-Fig. 4–top we show the 50th percentile (i.e., the median) as a function of probability. Unfortunately, in spite of our efforts to increase the experimental sensitivity, the measured success probability yielded zero for more than a half of the instances with $\tau = 10^5$ and $\tau = 10^6$. Hence, we show ED-Fig. 4–bottom the results for the 80th percentile.

In all cases, we found that a power law description

$$p(\tau, t_{\rm a}) \sim t_{\rm a}^{\theta(\tau)}\,, \tag{4}$$

is adequate, although the exponent θ depends significantly both on τ and on the percentile considered (see ED-Fig 5). The trends are very clear. For easy instances, p barely depends on $t_{\rm a}$ (yielding $\theta \approx 0$). In fact, the exponent θ increases with increasing τ, meaning that the harder the instance is, the more it typically benefits from increasing $t_{\rm a}$.

Recalling that time to solution is given by $t_{\rm s} = t_{\rm a}/p \sim t_{\rm a}^{1-\theta}$, we find that the exponent θ in Eq. (4) is less than 1 for all hardness groups, i.e., that the shorter the annealing time is, the shorter the time-to-solution becomes. Since however this trend can not hold all the way down to $t_{\rm a} = 0$, these results therefore imply that there exist for each group an optimal annealing time that is however below the shortest-accessible $t_{\rm a} = 20\mu$s. Furthermore, the increase of θ with hardness group can be interpreted as the harder the instances are, the longer the typical optimal annealing time is, consistently with what one would expect from a quantum annealer. It is important to note at this point that the highly-fluctuating success probability, stemming from programming errors, unfortunately does note allow for a more sensitive analysis and that more robust results could be obtained if the programing errors leading to J-chaos were to be reduced.

[1] R. Alvarez Baños, A. Cruz, L. A. Fernandez, J. M. Gil-Narvion, A. Gordillo-Guerrero, M. Guidetti, A. Maiorano, F. Mantovani, E. Marinari, V. Martin-Mayor, et al. (Janus Collaboration), J. Stat. Mech. **2010**, P06026 (2010), arXiv:1003.2569.

[2] M. E. J. Newman and G. T. Barkema, *Monte Carlo Methods in Statistical Physics* (Oxford University Press Inc., New York, USA, 1999).

[3] L. A. Fernandez, V. Martin-Mayor, and D. Yllanes, Nucl. Phys. B **807**, 424 (2009).

[4] A. P. Young, ed., *Spin Glasses and Random Fields* (World Scientific, Singapore, 1998).

[5] F. Barahona, T. Maynard, R. Rammal, and J. P. Uhry, J. Phys. A **15**, 673 (1982), URL `http://iopscience.iop.org/0305-4470/15/2/033`.

[6] H. G. Katzgraber, F. Hamze, and R. S. Andrist, Phys. Rev. X **4**, 021008 (2014), URL `http://link.aps.org/doi/10.1103/PhysRevX.4.021008`.

[7] T. F. Ronnow, Z. Wang, J. Job, S. Boixo, S. V. Isakov, D. Wecker, J. M. Martinis, D. A. Lidar, and M. Troyer, Science **345**, 420 (2014).

[8] T. Kadowaki and H. Nishimori, Phys. Rev. E **58**, 5355 (1998).

Extended Data

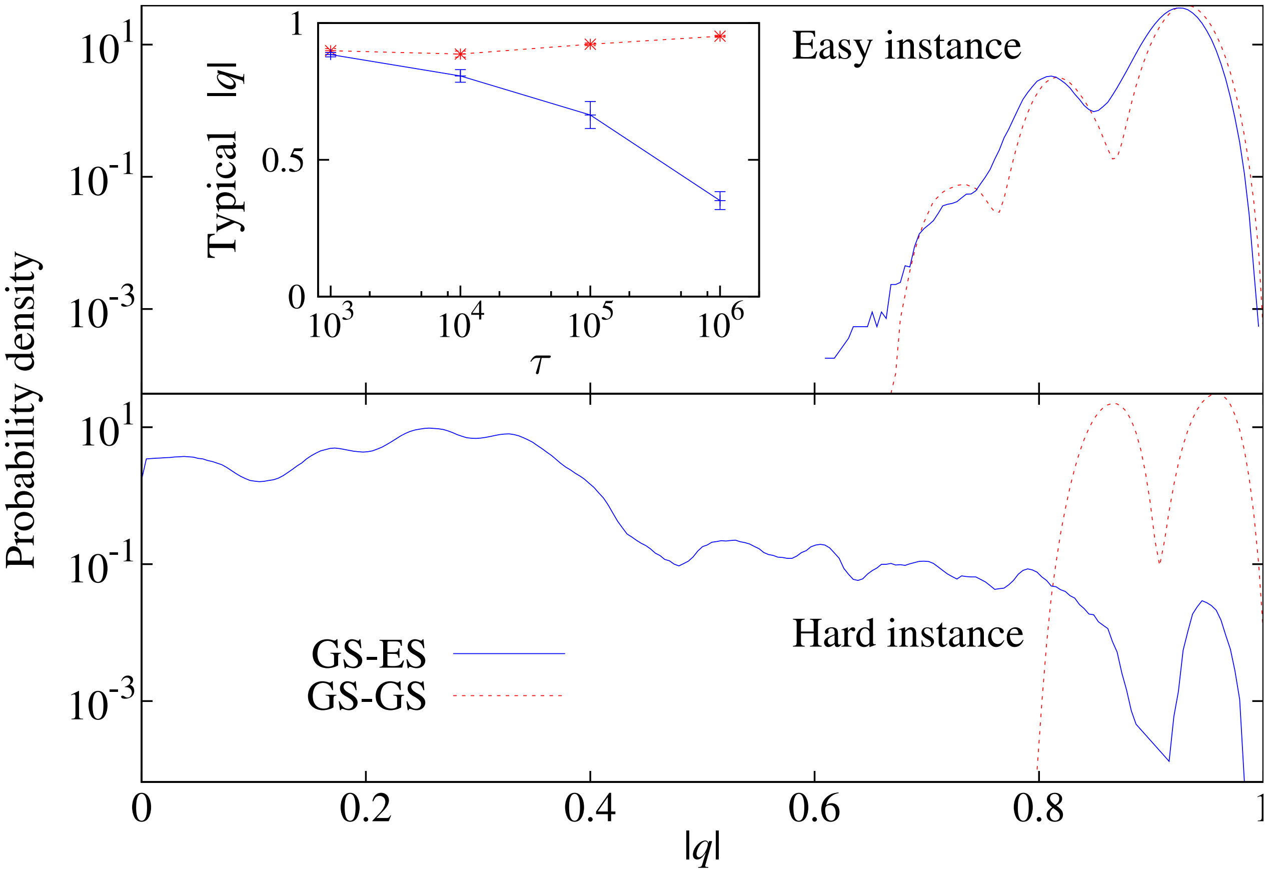

Extended Data Figure 1: **Probability distribution of the spin overlap $|q|$ between ground states and minimally excited states (GS-ES, solid blue curves).** For comparison, we also show the overlap between different ground states (GS-GS, dashed red curves). The distributions shown were computed for the same instances considered in Fig. 2 of main text, with mixing time generations $\tau \approx 10^3$ (**top**) and $\tau \approx 10^6$ (**bottom**). **Inset: Dependence of the overlap between ground states and minimally excited states on τ.** Typical median GS-ES overlap $|q|$ averaged over each hardness group as a function of τ-generation (blue points, the lines are to guide the eye).The red points are GS-GS typical overlaps shown for comparison.

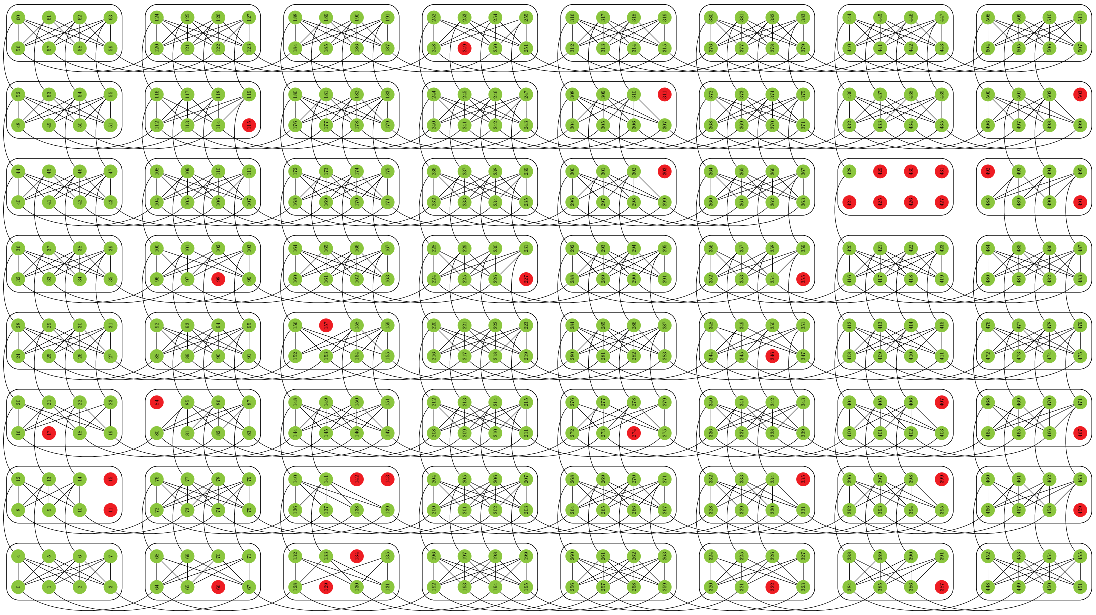

Extended Data Figure 2: **The 476-qubit DW2 device architecture and qubit connectivity.**

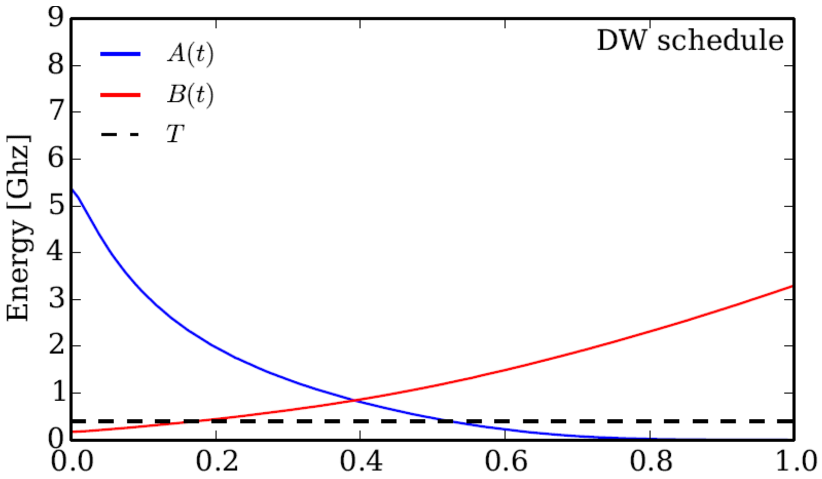

Extended Data Figure 3: **Annealing schedule of the D-Wave chip.** The functions $A(t)$ and $B(t)$ are the amplitudes of the (transverse-field) driver and classical Ising Hamiltonians, respectively. Also shown is the temperature in units of energy ($k_B = 1$).

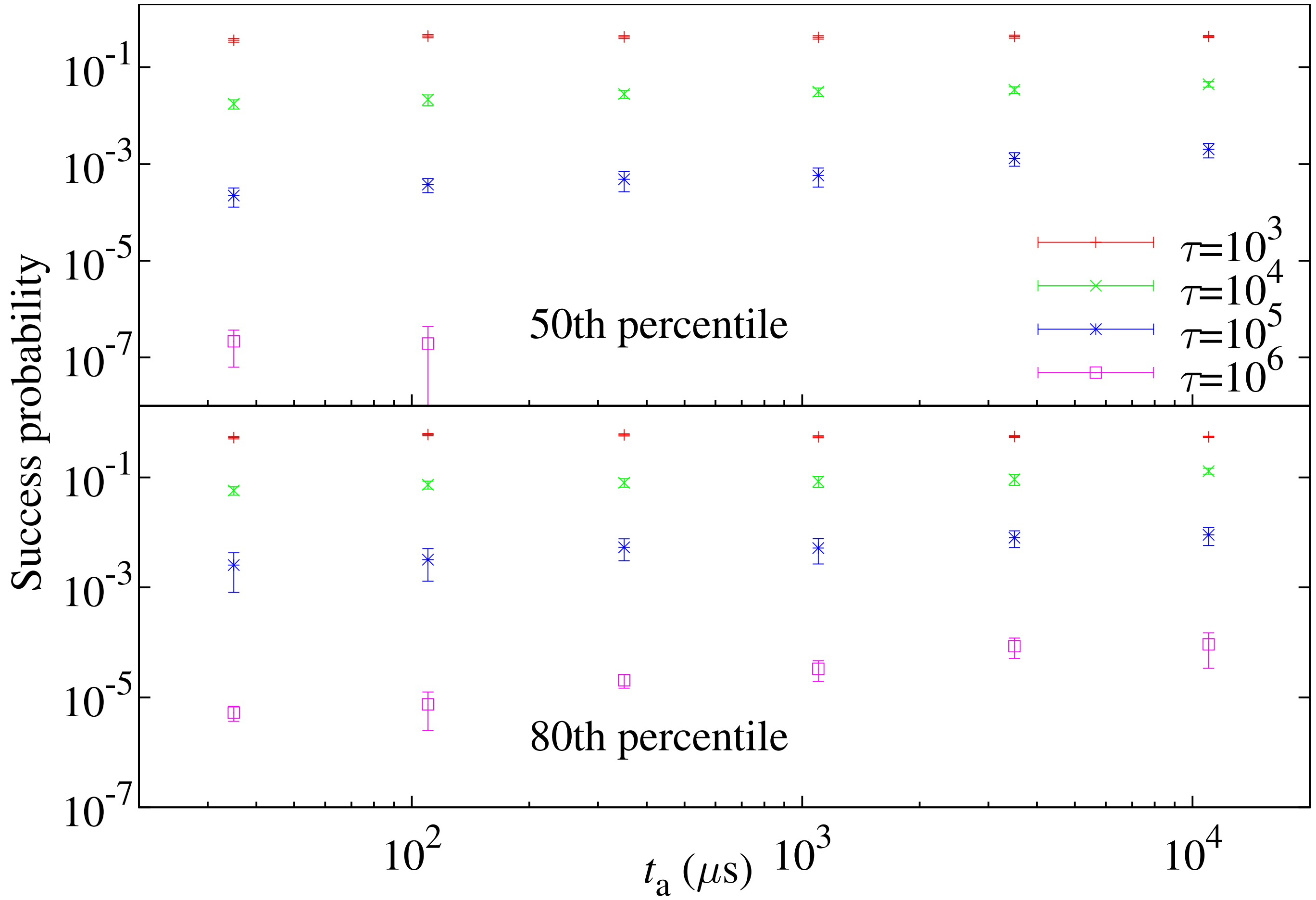

Extended Data Figure 4: **Dependence of success probability on annealing time.** Typical success probability (see main text for details) as a function of annealing time for the various hardness groups. Shown are the 50th and 80th percentiles within each hardness group (top and bottom panels, respectively).

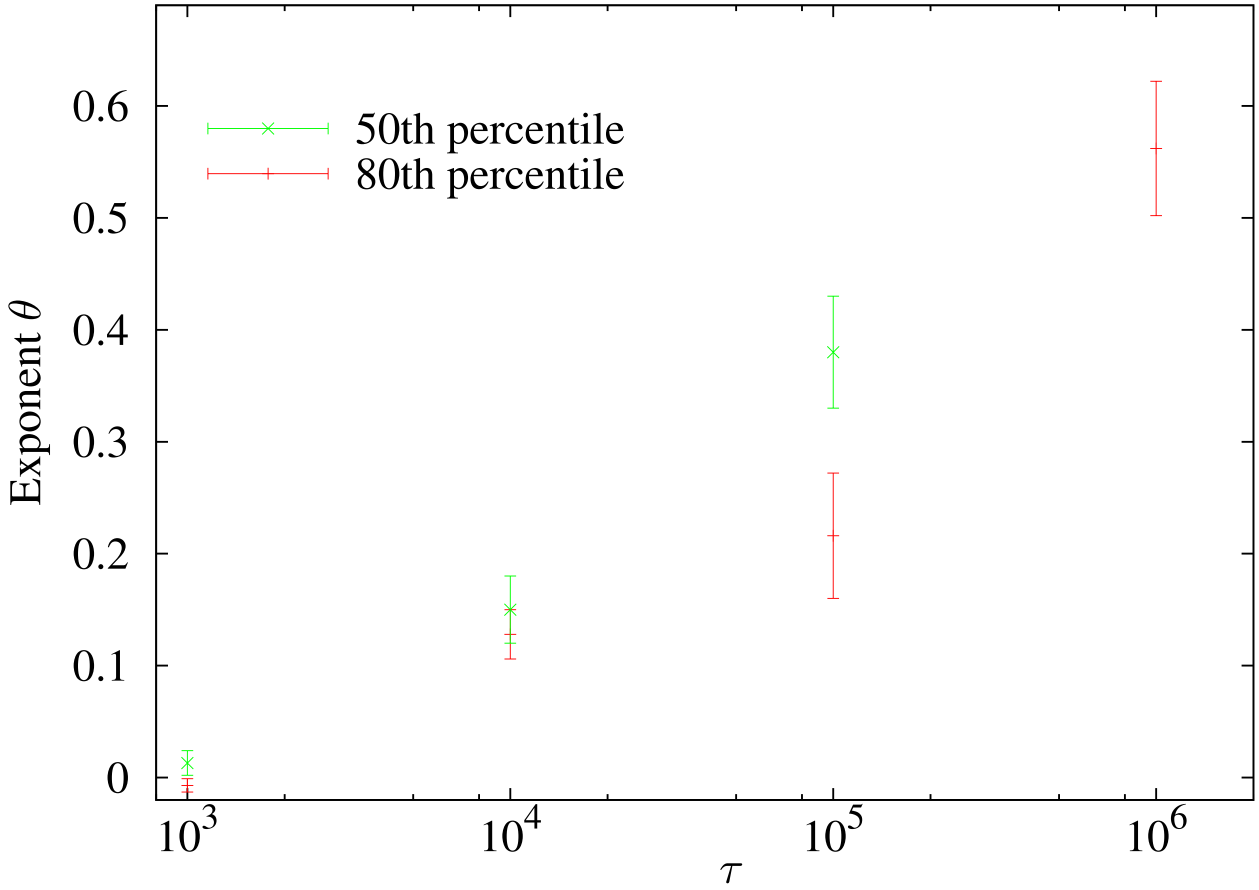

Extended Data Figure 5: **Dependence of success probability on annealing time.** The exponent θ of Eq. (4), as computed from the data shown in ED-Fig. 4, plotted against τ-generation.
